# Profil comparatif et évolutif des personnes infectées par le virus de l'immunodéficience humaine traitées aux antirétroviraux à Kinshasa, République Démocratique du Congo

**DOI:** 10.11604/pamj.2014.19.388.4287

**Published:** 2014-12-18

**Authors:** Tshingani Koy, Henri Mukumbi, Ghislain Lubangi Muteba Malandala, Philippe Donnen, Michèle Wilmet–Dramaix

**Affiliations:** 1 Centre de Recherche en Épidémiologie, Biostatistiques et Recherche Clinique, École de Santé Publique, Université Libre de Bruxelles, Bruxelles, Belgique; 2 Actions Communautaires Sida/ Avenir Meilleur pour les Orphelins au Congo (AMOCONGO), commune de Kasa Vubu, Kinshasa, RD Congo; 3 Université de Liège, Faculté de Médecine, Liège, Belgique

**Keywords:** Immunodéficience humaine, antirétroviraux, Kinshasa, trithérapie, human immunodeficiency, antiretroviral, Kinshasa, HAART

## Abstract

**Introduction:**

A trithérapie (ARV) introduite en R.D.Congo en 1996, a permis l′amélioration substantielle de la qualité de vie des PVVIH et a réduit la morbimortalité liée au sida en R.D. Congo. L'objectif de cette étude est de présenter le profil épidémiologique comparatif, clinique, ainsi que l’évolution anthropométrique des PVVIH sous ARV à Kinshasa.

**Méthodes:**

Etude de cohorte sur 438 PVVIH, de 18 ans et plus, suivies entre mai 2010 à 2011 à Amo Congo à Kinshasa. Une comparaison a été faite entre les patients suivis pendant un an et ceux perdus de vue. Le Chi carré de Mc Nemar et l'analyse de variance pour mesures répétées ont été appliqués pour étudier l’évolution.

**Résultats:**

Près 12 mois de suivi, 11,4% de patients ont été perdus de vue. Parmi eux, on observait des proportions significativement plus élevées de personnes de niveau socioéconomique bas, d'indice de masse corporelle (IMC) bas, présentant de l'anorexie, des affections opportunistes. Les proportions de patients aux stades OMS 3 & 4 et naïfs étaient également significativement plus élevées et la durée sous ARV plus courte. Les gains moyens des paramètres anthropométriques au 12ème mois, étaient importants: de 3,6 [3,2 - 4,0] kg pour le poids, 1,8 (1,4 - 2,3) cm pour le périmètre abdominal, 0,9 (0,8 - 1,2) cm pour le périmètre brachial, 1,4 (1,2 - 1,5) kg/m^2^ pour l'IMC. La proportion de patients avec un IMC <18,5 kg/m^2^ a significativement plus baissé entre l'admission et le 12^ème^ mois parmi les patients sans stomatite que parmi ceux avec stomatite. L'IMC moyen évoluait significativement différemment entre l'admission et le 12^ème^ mois selon l’âge et la taille de ménage.

**Conclusion:**

Les facteurs fragilisant la rétention des patients sous antirétroviraux et une évolution progressive de l’état nutritionnel ont été observés.

## Introduction

Dans les pays les plus touchés par le VIH/SIDA, les conséquences de cette épidémie dépassent le domaine de la santé et affectent également le développement socioéconomique, la sécurité alimentaire et la nutrition. D'après le rapport de l'institut de recherches de la politique alimentaire internationale (IFPRI) en 2013, l'indice global de la famine (GHI) qui présente les mesures multidimensionnelles de la famine sur le plan global, régional et national, a montré que le niveau de la faim dans le monde avait chuté d'un tiers depuis 1990. Néanmoins, l'Asie du Sud et l'Afrique subsaharienne restent les régions planétaires qui ont les niveaux de faim les plus élevés [1]. Des efforts considérables sont réalisés en Afrique pour réduire ce GHI par des progrès notamment en matière de stabilité politique et économique de certaines nations ainsi que par certaines améliorations en matière de santé: l'accès à l'eau saine et potable, l'immunisation, la lutte contre le paludisme et le sida [1], néanmoins la prévalence du VIH/SIDA reste aussi élevé dans ce même continent africain. Le lien entre la malnutrition et le VIH a déjà été étudié dans le passé. En effet, la faim modifie les comportements des individus, affaiblit leurs corps et leurs systèmes immunitaires, et les exposent à des vulnérabilités aux infections dont le VIH, le paludisme et la tuberculose. Raison pour laquelle, plusieurs programmes nationaux de lutte contre le VIH/SIDA ont adopté des stratégies de prise en charge globale, associant à la prise en charge thérapeutique, l'accompagnement psychosocial et la prise en charge nutritionnelle. Différents programmes s'efforcent d'améliorer ou de renforcer leurs politiques aussi bien sur la qualité de l′alimentation que sur les apports alimentaires quantitatifs pour offrir aux populations une alimentation couvrant les seuils recommandés dans des régions où la malnutrition est endémique [2].

En ce qui concerne la République démocratique du Congo (RDC) en 2013, à cause des conflits armés, obstacles évidents à l'amélioration de la situation, l'index GHI n'a pas pu être calculé et ne pourra l’être que dans le futur [1]. Néanmoins, en 2011 elle était classée dans la catégorie extrêmement alarmante [1], et bien avant, d'autres études ont fait l’état de lieu de la sécurité alimentaire en R.D C. Les études effectuées en 2003 [3] et en 2008 [4] n'attestent pas non plus d'une progression nette de la situation nutritionnelle sur l'ensemble du territoire congolais. Cependant, la malnutrition reste également un problème préoccupant pour les personnes vivant avec le VIH (PVVIH) en RD Congo. L′avènement de la trithérapie en 1996, a permis d′améliorer de façon substantielle la qualité de vie des PVVIH et de réduire la morbimortalité liée au sida. Mais en pratique clinique, la prise en charge incluant les antirétroviraux (ARV) reste une prise en charge à vie, ayant aussi bien des effets thérapeutiques bénéfiques que des effets secondaires parmi lesquels des troubles métaboliques importants notamment la lipodystrophie, la résistance à l'insuline et le changement des concentrations plasmatiques des lipides et des lipoprotéines [5, 6]. Et la littérature a déjà évoqué les conséquences de la présence virale dans le sang sur l'accroissement des dépenses énergétiques, provoquant ainsi une protéolyse musculaire avec une perte exagérée de tissu maigre des PVVIH [7]. Bien plus, pour atteindre un succès clinique, immunologique et virologique post antirétroviral, une bonne observance, compliance et un apport nutritionnel équilibré et régulier sont exigés aux patients sous ARV. Cette étude a été menée en tenant compte de toutes les exigences évoquées ci haut, auxquels s'associent les possibilités qui sont données aux PVVIH de rester dans le système de soins, pour avoir un succès de la prise en charge. Elle a eu pour objectif de présenter le profil épidémiologique, clinique, ainsi que l’évolution anthropométrique des PVVIH suivies sous antirétroviraux dans trois différents sites du centre de traitement ambulatoire (CTA/ AmoCongo) de Kinshasa.

## Methode

### Population d’étude et critères d'inclusion

Il s'agit d'une étude de cohorte prospective sur 438 PVVIH suivies à Kinshasa entre mai 2010 et mai 2011. Parmi les critères d'inclusion, nous avons retenu les patients âgés de 18 ans et plus traités aux ARV. Les femmes enceintes sous ARV ont été exclues de cette étude.

### Sites d’étude et collecte des données

L’étude a été menée dans trois différents sites du CTA ACS/AmoCongo de Kinshasa: Kasa Vubu, Mbinza Ozone et Ndjili. Ces sites étaient inscrits dans une approche globale de prise en charge: le dépistage volontaire du VIH, la prise en charge médicale et psychosociale des PVVIH. Les données ont été collectées lors du suivi des malades à l'admission, au troisième, sixième et douzième mois. Les variables collectées étaient l’âge, l’état civil, le cadre de vie, la taille de ménage, le niveau d’étude et le statut socioéconomique. Ces variables ont été catégorisées: l’âge dichotomisé en moins de 50 ans et supérieur ou égal à 50 ans en accord avec la littérature [8], l’état civil en célibataire, marié (e), divorcé(e) et veuf(ve), le cadre de vie en vivant seul, en famille chez soi ou chez les tiers (un parenté), la taille de ménage en pas d'enfant, 1 à 3 enfants, 4 enfants ou plus; le niveau d’étude en niveau bas (maximum primaire), moyen (secondaire), élevé (Supérieur ou Universitaire). Un score fait du revenu mensuel, les biens matériels des patients, l'activité professionnelle réelle, a été crée d'une somme simple allant de 0 à 30. Et il a permis de catégoriser les patients en niveau socioéconomique bas pour le tertiles inférieur, moyen pour le tertiles moyen et élevé pour le tertiles supérieur. Les données anthropométriques ont été collectées: la station de pesée et de mesure de marque SECA a été utilisée pour collecter le poids en kg et la taille en cm, le périmètre brachial en centimètre (cm) et le périmètre abdominal en cm au moyen d'un mètre ruban. Nous avons recueilli les signes, ainsi que certains diagnostics cliniques suivants: l'anorexie, l'asthénie, la stomatite, l'autonomie d'activité, la présence d'affections opportunistes, le statut du patient par rapport aux ARV(les patients naïfs et les non naïfs aux ARV). Le stade clinique du sida selon l'OMS a été dichotomisé en stade du début regroupant le stade 1 & 2, et le stade avancé incluant les stades 3 & 4. Parmi les paramètres biologiques, nous avons collecté: l'hémoglobine en g/dl dichotomisé en inférieur et supérieur ou égal à 12 g/dl et le nombre de CD4 en inférieur et supérieur ou égal à 250 cellules/µl.

### Analyse statistique

Pour décrire l’échantillon, une comparaison a été faite entre les patients qui ont été suivis pendant un an et ceux qui ont été perdus de vue durant le suivi. Le test t-student et l'analyse de variance à un facteur ainsi que le Chi carré de Pearson ont été utilisés pour comparer les moyennes et les proportions. Pour l’évolution de l’état nutritionnel durant le suivi, le Chi carré de Mac Nemar a été appliqué de même que l'analyse de variance pour mesures répétées. Les analyses ont été réalisées au moyen du logiciel Open Epi version 2.3.1 et SPSS version 20.

## Resultat

### Caractéristiques des malades inclus à l'admission

Au total 438 PVVIH ont été incluses dans cette étude au mois de mai 2010. Après un suivi d'une année 11,4% des malades ont été perdus de vue. L’âge moyen des malades ne différait pas significativement entre le groupe des suivis et ceux perdus de vue. On notait aussi dans les deux groupes une proportion élevée de femmes, de malades mariés, et de malades vivant sous le toit familial. Les malades étaient en majorité d'un niveau d’étude moyen. Les malades avec un niveau socioéconomique bas étaient significativement plus fréquents parmi les malades perdus de vue (53,1%) que chez ceux suivis un an (31,5%) (P < 0,008). La proportion de malades malnutris (IMC < 18,5 kg/m^2^) était significativement plus élevée dans le groupe des malades perdus de vue que dans le groupe de ceux suivis pendant un an (32,0% versus 20,1%, P < 0,001) (Tableau 1). Les proportions de signes cliniques: anorexie (48,0% versus 32,5%, P= 0,029), la présence d'affection opportuniste (48,0% versus 19,8%, P < 0,001), le stade clinique avancé du sida (87,8% versus 76,4%, P= 0,017) étaient significativement plus élevées parmi les malades perdus de vue que parmi les malades suivis pendant un an. La proportion de malades naïfs était significativement plus élevée chez les perdus de vue que parmi les malades suivis pendant 1 an. La durée moyenne des patients sous ARV était significativement plus élevée chez ceux qui ont été suivis pendant un an 3,0 (2,7) versus 2,0(1,5) (P < 0,001) (Tableau 2). Les proportions des différents types d'affections opportunistes ne différaient pas significativement entre les deux groupes. La majorité des patients était traitée aux ARV de première ligne (zidovudine, lamivudine, névirapine).

**Tableau 1:** Variables cliniques à l'admission des PVVIH suivies pendant un an et de celles perdues de vue au cours du suivi

Variables	Suivis pendant 1 an (n = 388)		Perdus de vue(n = 50)		P
	Moy (DS)	%	Moy (DS)	%	
1. Age en année	42,0 (9,3)	-	40,9 (9,9)	-	NS
< 50	-	76,5	-	76	NS
2. Sexe					
Femme	-	72,7	-	82	NS
3. Etat civil					
Célibataire	-	27,8	-	26	NS
Marié(e)	-	34,3	-	40	-
Divorcé(e)	-	10,8	-	12	-
Veuf (ve)	-	27,1	-	22	-
4. Cadre de vie					
Vit seul	-	3,9	-	2	NS
Vit chez soi	-	75	-	66	-
Vit chez les tiers	-	21,1	-	32	-
5. Taille de ménage					
Pas d'enfant	-	34	-	30	NS
1-3 enfants	-	29,1	-	32	-
≥ 4 enfants	-	36,9	-	38	-
6. Niveau d’étude					
Niveau bas	-	8,5	-	14	NS
Niveau moyen	-	66,2	-	68	-
Niveau élevé	-	25,3	-	18	-
7. Niveau SE*					
Bas	-	31,5	-	53,1	<0,08
Moyen	-	48,5	-	28	-
Aisé	-	20,1	-	20	-
9 Poids (Kg) (n)	59,4(12,0)	-	54,9 (12,5)	-	0,005
10. IMC* (Kg/m^2^)(n)	22,1(4,2)	-	19,9 (4,0)	-	<0,001
<18,5	-	-	-	-	-
≥18,5	-	-	-	-	-
11. PAbd*(cm)(n)	79,6 (9,4)	-	74,0 (9,9)	-	<0,01
12. PBr*(cm)(n)	26,2(4,0)	-	25,0(4,3)	-	NS

* SE= Socioéconomique

* IMC = Indice de masse corporelle

* P.Abd= Périmètre abdominal

* P.Br = Périmètre brachial

**Tableau 2:** Variables cliniques à l'admission des PVVIH suivies pendant un an et de celles perdues de vue au cours du suivi

Variables	Suivis pendant 1n (n = 388)		Perdus de vue (n = 50)		P
	Moy(DS)	%	Moy(DS)	%	
**1. Stomatite**					
Oui	-	9,5	-	18	NS
Non	-	90,5	-	82	-
**2. Anorexie**					
Oui	-	32,5	-	48	0,029
Non	-	67,5	-	52	-
**3. Affection opportuniste(OP)**					
Oui	-	19,8	-	48	<0,001
Non	-	80,2	-	52	-
**4. Type d'affection OP (n = 76)**				**(n = 23)**	
Affections digestives	-	35,4	-	31,8	NS
Affections pulmonaires	-	40,8	-	50	-
Affections dermatologiques	-	18,4	-	22,7	-
Autres affections	-	5,2	-	4,5	-
**5. Activité physique autonome**					
oui	-	57,4	-	42	0,039
non	-	42,6	-	58	-
**6. Statut du patient aux ARV**					
patients naïfs	-	13,9	-	51	<0,001
patients non naïfs	-	86,1	-	49	-
**7. Durée sous ARV (en année)**	3,0 (2,7)	-	2,0 (1,5)	-	<0,001
8. Régime d'ARV	-	-	-	-	-
AZT + 3TC + NVP	-	87,4	-	85,7	<0,001
AZT + 3TC + EFV	-	7,2	-	12,2	-
ABC + DDI + KALETRA	-	5,4	-	2	-
**9. Stade OMS/ SIDA (n = 386)**				**(n = 50)**	
Stade débutant (1&2)	-	23,6	-	12,2	0,017
Stade avancé (3& 4)	-	76,4	-	87,8	-
**10. CD4 (cellule/µl) (n = 74)**				**(n = 23)**	
<250	-	50	-	47,8	NS
≥250	-	50	-	52,2	-
**10. CD4 (cellule/µl) (n = 74)**				**(n = 23)**	
<250	-	50	-	47,8	NS
≥250	-	50	-	52,2	-

AZT = Zidovudine KALETRA = Lopinavir & Ritonavir 3TC = Lamivudine ABC= Abacavir NVP= Névirapine DDI= Didanosine EFV= Efavirenz

### L’évolution des malades après une année de suivi

Après une année de suivi, on a observé une augmentation statistiquement significative du poids, périmètre abdominal, périmètre brachial, et de l'indice de masse corporelle. Les différences des moyennes des poids, périmètre abdominal, périmètre brachial et de l'indice de masse corporelle étaient statistiquement significatives entre l'admission et le 12ème mois. Les gains moyens étaient les plus importants au 12ème mois (Tableau 3). On observait aussi une diminution significative des proportions de patients avec un indice de masse corporelle inférieur à la normale. La proportion de patients avec un IMC inférieur à 18,5 kg/m^2^ a significativement diminué à 9,6% au 12 ^ème^ mois (Tableau 4). L'analyse de l’évolution des proportions de patients qui avaient un IMC bas (<18,5 kg/m^2^) à l'admission et qui ont atteint un bon IMC (≥18,5 kg/m^2^ ) au 12^ème^ mois en fonction des caractéristiques sociodémographiques, socioéconomiques et cliniques, a permis d'observer des proportions significativement plus élevée d'amélioration de l'IMC parmi les patients n'ayant pas présenté la stomatite à l'admission par rapport à ceux qui ont eu la stomatite. Sur les 54 patients sans stomatite avec un IMC < 18,5 kg/m^2^, 63,0% soit 34 patients ont atteint un IMC ≥ 18,5 kg/m^2^. Par contre chez ceux qui ont présenté la stomatite avec un IMC < 18,5kg/m^2^, sur 24 patients à l'admission, 8 patients seulement ont atteint un IMC ≥ 18,5 kg/m^2^ au 12^ème^ mois (soit 33,3%). Nous n'avons pas observé de différences significatives des proportions des patients ayant atteint un IMC ≥18,5 kg/m^2^ au 12^ème^ mois en fonction des autres caractéristiques des patients cependant en l'absence des signes cliniques, les proportions de retour à un BMI normal étaient en général plus élevées (Tableau 5). L'analyse de l'effet des caractéristiques sociodémographiques, socioéconomiques et cliniques des patients à l'admission sur la différence des moyennes de l'IMC entre l'admission et le 12^ème^ mois, a montré des évolutions significatives des moyennes de l'IMC en fonction de l’âge et de la taille de ménage. En effet, les patients de plus de 50 ans avaient une différence de gain moyen de l'IMC significativement plus basse que ceux de moins de 50 ans. Et les patients ayant au moins un enfant présentaient une différence de gain des moyennes de l'IMC significativement inférieure par rapport à ceux n'ayant pas d'enfant. Les autres caractéristiques n'ont pas présenté d'interaction cet à dire des modifications des performances d’évolution au cours au cours du temps de suivi.

**Tableau 3:** Analyse de l’évolution des mesures anthropométriques des PVVIH suivies pendant un an, entre l'admission et le 12^ème^ mois

Variables	Admission (n = 386)	3^ème^ mois (n = 386)	6^ème^ mois (n = 386)	12^ème^mois (n = 386)	P
	Moy (DS)	Moy (DS)	Moy (DS)	Moy (DS)	
**Poids (kg)**	59,4 (12,0)	59,5 (11,9)	60,9 (12,0)	63,0 (12,2)	<0,001
Diff [IC 95%]	-	0,2 [0,0 - 0,4]	1,6 [1,3 - 1,9]	3,6 [3,2 - 4,0]	
**P.Abd (cm)**	79,7 (9,3)	80,4 (9,3)	80,9 (9,3)	81,5 (9,5)	<0,001
Diff [IC95%]	-	0,8 [0,5 - 1,0]	1,2 [0,9 - 1,6]	1,8 [1,4 - 2,3]	
**P.Br (cm)**	26,2 (4,0)	26,3 (3,9)	26,7 (3,8)	27,2(3,8)	<0,001
Diff [IC 95%]	-	0,1 [0,0 - 0,2]	0,5 [0,4 - 0,7]	0,9 [0,8 - 1,2]	
**IMC (kg/m^2^)**	22,1 (4,2)	22,7 (4,2)	23,5 (4,3)	23,5(4,3)	<0,001
Diff [IC 95%]	-	0,6 [0,0 - 0,2]	1,4 [0,5 - 0,7]	1,4 [1,2 - 1,5]	

IMC= Indice de masse corporelle P.Abd= Périmètre abdominal P. Br= Périmètre brachial Diff [IC 95%]= Différence [Intervalle de confiance à 95%]

**Tableau 4:** Évolution des proportions des patients avec IMC < 18,5 kg/m^2^ entre l'admission et le 12^ème^ mois

Variables	N total = 386										
IMC < 18,5kg/m^2^	**N**	**%**	**P**	**-**	**N**	**%**	**P**	**-**	**N**	**%**	**P**
Admission	78	20,2	<0,001	Admission	78	20,2	<0,001	6^ème^Mois	61	15,8	<0,001
6^ème^ Mois	61	15,8		12^ème^Mois	37	9,6		12^ème^Mois	37	9,6	

**Tableau 5:** Proportions des patients avec IMC ≥ 18,5 kg/m^2^ à 12 mois parmi les patients avec IMC <18,5 kg/m^2^ à l'admission en fonction des variables sociodémographiques, socioéconomiques et cliniques

Variables	IMC < 18,5 kg/m^2^ à l'admission n(%)	IMC < 18,5 kg/m^2^ au 12^ème^ moisn(%)	P
**Age (années)**			
< 50	63 (21, 2%)	32 (50, 8%)	0,26
≥ 50	15 (16, 5%)	10 (66, 7%)	
**Taille de ménage**			
Sans enfant	34 (25, 8%)	15 (44,1%)	0,13
Au moins un enfant	44 (17, 2%)	27 (61,4%)	
Niveau SE*			
bas	32 (26,2%)	16 (50,0%)	0,57
Moyen & élevé	46 (17,3%)	26 (56,5%)	
**Stomatite**			
Non	54 (15,4%)	34 (63, 0%)	0,015
Oui	24 (64,9%)	8 (33, 3%)	
**Anoréxie**			
non	25 (9,5%)	16 (64, 0%)	0,21
Oui	53 (42,1%)	26 (49, 1%)	
**Affection opportuniste**			
non	51 (16,4%)	27 (52, 9%)	0,82
Oui	27 (35,1%)	15(55, 6%)	
**Asthénie physique**			
non	28(10, 3%)	19 (67, 9%)	0,06
Oui	50(43,9%)	23 (46, 0%)	
**Stade clinique du Sida**			
1&2	3 (3, 3%)	2 (66, 7%)	0,65
3&4	75 (25, 4%)	40 (53, 3%)	
**Autonomie d'activité physique**			
Non	11 (5,0%)	7 (63, 6%)	0,48
Oui	67 (40,6%)	35 (52,2%)	

* SE= socioéconomique

## Discussion

Cette étude avait pour objectif de présenter le profil épidémiologique, clinique, ainsi que l’évolution anthropométrique des patients sous ARV suivis dans les sites du CTA d′Amocongo de Kinshasa. Les réalités vécues lors des recherches menées sur terrain confirment la déclaration selon laquelle les faibles moyens d'investigation clinique, para clinique ainsi que les faibles moyens économiques des populations, réduisent l'efficacité des interventions visant à corriger les déficiences nutritionnelles chez les PVVIH et redynamiser la prise en charge dans les pays en développement [9]. Néanmoins, les recherches continuent dans le but d'identifier des interventions qui visent à trouver des solutions adéquates pour améliorer aussi bien l’état nutritionnel que la prise en charge. Certains auteurs estiment par exemple, qu'il est important lors de ces interventions nutritionnelles, de tenir compte aussi bien de l’évaluation de l’état nutritionnel, des conseils et soutien nutritionnels et notamment de l'administration des suppléments nutritionnels que des moyens de subsistance des patients [10–12]. Cette étude s'inscrit dans la lignée de celles qui recherchent les facteurs qui améliorent la rétention des patients dans le circuit de soins et porte aussi un regard sur l’évaluation de l’état nutritionnel des patients atteints du VIH traités dans le milieu congolais. L'arrivée des ARV a contribué efficacement au soulagement des effets néfastes du virus en réduisant la charge virale, les infections opportunistes et aussi en réduisant la potentialisation de la perte énergétique liée au métabolisme de base des patients infectés par le virus. Pour un résultat optimum attendu des ARV, une bonne compliance, une bonne observance, ainsi qu'un bon accompagnement psychosocial, notamment nutritionnel des patients, doivent être maintenus. Au cours de cette étude nous avons observé un pourcentage de 11,4% des patients perdus de vue (patients qui n'ont pas été revus au rendez-vous de suivi du traitement aux ARV, au cours d'un suivi de 12 mois). Parmi ces patients, nous avons observé une proportion élevée de patients avec un niveau socioéconomique bas, et une moyenne d'IMC basse; on a noté aussi parmi eux, des proportions élevées de plaintes d'anorexie, de présence d'affections opportunistes, et d'absence d'autonomie des activités physiques de la vie quotidienne. Des proportions élevées de patients avec un stade clinique du sida élevé (3 & 4) ainsi que de patients naïfs (les nouveaux admis au traitement) ont été également observées aussi parmi les perdus de vue. Cependant, dans notre étude, la proportion de perdus de vue était faible par rapport à celle observée dans d'autres études. Rosen et al [13], lors d'une revue systématique, ont obtenu une proportion de 25% de perdus de vue après 12 mois de suivi; Matthew P et al [14] ont obtenu 59% pour des suivis de 12, 24 et 36 mois, dans des études menées en Afrique subsaharienne entre 2007-2009. Plusieurs études en sciences sociales et médicales ont identifié comme cause entre autres d'abandon de traitement: les coûts associés, le moyen de transport et le temps d'attente, la stigmatisation, la pression familiale, les croyances religieuses et la maladie elle-même [13, 15]. Dans cette étude, néanmoins, nous avons observé deux facteurs qui exposent les patients à un abandon de traitement: c'est notamment la pauvreté et le stade de la maladie. En effet, sans un soutien financier qui appuie l'amélioration des conditions socioéconomiques, base d'un bon suivi des soins, le long chemin de prise des ARV devient très vite infranchissable. Bien plus, nous avons observé que la proportion de patients avec les stades cliniques avancés 3&4 étaient plus élevées dans ce groupe des perdus de vue.

Le stade clinique indique l’état d'avancement de la maladie. Il est un facteur de vulnérabilité des patients face aussi bien à la maladie elle-même, aux affections opportunistes qu'au long et contraignant chemin thérapeutique des ARV. Toujours parmi les perdus de vus, nous avons noté une proportion plus élevée de patients avec un mauvais état nutritionnel. Les études antérieures ont révélé qu'aux stades très avancés du sida on assistait à une réduction des apports nutritionnels, une augmentation des dépenses énergétiques liées au métabolisme basal due à la charge virale [7, 16, 17]. Cette présence virale déclenche une protéolyse qui entraine des lésions musculaires causant ainsi un déséquilibre de la balance en nitrogène et une perte exagérée de tissu maigre [7]. D'autre part, on assiste, à une diminution des apports alimentaires, entraînant un déséquilibre de la balance pondérale, de l'asthénie physique et une incapacité du patient à réaliser les activités physiques liées aux besoins de la vie quotidienne [18, 19] voire même de rechercher les soins. Ceci peut justifier la perte au cours du suivi des patients plus vulnérables. D'une manière générale, nous avons observé dans notre échantillon, une proportion élevée de femmes (> 72%) parmi les PVVIH ainsi qu'une moyenne d’âge de 40 ans. Cette observation concorde avec celle d'autres études [14, 20]. Ainsi donc, nous plaidons pour un renforcement des mesures des préventions sur le VIH /SIDA auprès des populations cibles en général et des femmes en particulier ainsi qu’à la réduction des vulnérabilités qui les exposent à la maladie. Un dépistage précoce ainsi qu'une prise en charge thérapeutique de ceux qui sont atteints et un accompagnement psychosocial précoce en général et dans la tranche d’âge de moins de 50 ans en particulier doivent être de principe. Après une année de suivi des patients sous ARV, nous avons observé une évolution positive des moyennes des paramètres anthropométriques des patients. Les gains moyens IC à 95% étaient les plus importants au 12^ème^ mois et ils étaient respectivement de 3,6 (3,2 - 4,0) kg pour le poids, 1,8 (1,4 - 2,3) cm pour le périmètre abdominal, 0,9 (0,8 - 1,2) cm de périmètre brachial, et 1,4 (1,2 - 1,5) kg/m^2^ de l'indice de masse corporelle. L'usage des paramètres anthropométriques dont notamment l'IMC comme outil du monitoring du pronostic mais aussi comme marqueur de l'efficacité du traitement ARV a été décrit et demeure important dans les milieux à ressource limitée [21, 22]. L’évolution du périmètre brachial et abdominal est tout aussi importante car elle complète l’évaluation non seulement de l’état de santé du patient mais aussi de l'efficacité du traitement dont notamment la surveillance des troubles métaboliques qui peuvent être imputés aux ARV. Bien qu’étant positive, l’évolution de l'IMC ainsi que celle du poids des patients dans cette étude nous semblent être faible par rapport à celle des patients suivis en Afrique du Sud [23].

Après 12 mois de suivi, le gain moyen de l'IMC (IC à 95%) des patients Sud Africains, était respectivement de 2,4 (1,7-3,1) (kg/m^2^) chez les hommes et de 2,2 (1,5-2,9) (kg/m) ^2^ chez les femmes et ce gain était associé à l’âge. Le poids moyen (DS) des patients Sud Africains était de 63,6 (2,1) kg et 63,5 (2,5)kg avec un gain de 6,8 (4,9- 8,7) kg et 5,6 (3,8-7,3) kg respectivement chez les hommes et chez les femmes après 12 mois de suivi alors que dans notre étude le poids moyen global était de 59,4 (12,0) kg avec un gain pondéral de 3,6 (3,2 - 4,0) kg. Cette différence marquée peut s'expliquer par le niveau socioéconomique des patients, qui est très différent dans les deux échantillons: il est plus élevé parmi les patients sud africains (58% d'un niveau aisé versus 20% dans notre étude); bien plus, dans beaucoup de milieux africains, la notion de la perception de la taille et poids corporel idéal faisant parti des normes culturelles, une perte importante du poids est mal vécue car, marqueur externe de la maladie et de la stigmatisation entourant le VIH. Cette perception, qui reste réelle aussi pour d'autres milieux africains, a été observée dans l’étude Sud-africain [23]. Les patients (PVVIH) sud africains qui souhaitaient prendre du poids, avaient modifié leurs régimes diététiques et mode de vie, et avaient considérablement augmenté leur poids [23]. Cette notion de perception de soi et le gain pondéral parmi les PVVIH doivent être étudiés en profondeur dans le milieu Congolais. Nos résultats sur le gain pondéral par contre, sont légèrement supérieur à ceux d'une autre étude menée dans un milieu à ressource limitée en côte d'ivoire [22] où les chercheurs ont observé une évolution significative de l'IMC dont le gain moyen a été de 0,7 kg/m^2^ après 6 mois de suivi chez les patients avec un IMC moyen (DS) de départ de 22,5 (2,5) kg/m^2^ alors que dans nos résultats, le gain moyen était de 1,4 (0,5 ≥ 0,7) kg/m^2^ à 6 mois pour l'IMC moyen (DS) à l'admission de 22,7 (4,2). Globalement, la proportion de patients avec un IMC moyen inférieur à 18,5 kg/m^2^ à l'admission a significativement diminué jusqu’à 9,6% au 12^ème^ mois. Néanmoins, parmi des patients avec IMC < 18,5 kg/m^2^, nous avons observé une proportion significativement plus élevé de l'IMC ( ≥18,5 kg/m^2^) entre l'admission et le 12^ème^ mois, parmi les patients n'ayant pas présenté la stomatite que ceux qui en présentaient. L'absence de stomatites au cours du sida est bénéfique pour une bonne récupération de l’état nutritionnel. La présence des lésions des muqueuses buccales contribue à la diminution des apports alimentaires par l'anorexie, un des plus importants facteurs de risque clinique de la malnutrition [24]. Et la persistance de certaines lésions des muqueuses notamment la candidose buccale sont associées avec un échec immunologique et une progression du sida [25].

À la fin du suivi, le gain moyen de l'IMC différait significativement seulement en fonction de deux caractéristiques sociodémographiques: les patients âgés d'au moins 50 ans ont présenté un gain moyen l'IMC significativement inférieur à celui de moins de 50 ans; et ceux ayant au moins un enfant, une différence de gain moyen d'IMC significativement inférieure par rapport à ceux n'ayant pas d'enfant. À l'admission dans l’étude les patients âgés d'au moins 50 ans présentaient une moyenne d'IMC supérieure à celle de moins de 50 ans, et notons que dans cette échantillon, parmi les patients de 50 ans et plus, on notait un grand nombre de patients à un stade clinique débutant ce qui corrobore avec l'histoire du profil épidémiologique des PVVIH en RDC [18, 26]. En effet, La littérature évoque que dans cette tranche d’âge, en plus du disfonctionnement immunitaire qui est lié au virus du sida, l'organisme subit des défis liés au vieillissement du système immunitaire, qui exposent aux infections secondaires, et à une réponse encore plus lente du système immunitaire [8, 27]. Bien plus, les patients qui présentent un dysfonctionnement immunitaire en général et ceux de plus de 50 ans en particulier répondent plus faiblement aux traitements antirétroviraux, et présentent des problèmes de réplication virale et des comorbidités au VIH [28]. Avec l'avènement des ARV, l'espérance de vie des patients a été considérablement améliorée. L’âge avancé des patients sidéens devient un problème de santé publique actuellement et suscite une attention particulière des prestataires. Les patients ayant au moins un enfant ont présenté un gain moyen d'IMC inférieur par rapport à celui des patients n'ayant pas d'enfant à la fin du suivi. Le revenu du ménage joue un grand rôle dans la prise en charge nutritionnelle des familles. En effet, dans le contexte des pays à ressources limitées, l'entrée du sida dans un ménage est source de stress et de beaucoup de problèmes financiers liés aux dépenses des soins médicaux. Elles s'ajoutent aux dépenses liées aux besoins fondamentaux pour les ménages et réduisent les possibilités d'accès à un régime alimentaire équilibré et suffisant pour le ménage. Cette situation est encore d'autant plus dramatique que la taille du ménage est très large et peut être à la base d'une insécurité alimentaire du ménage. Ceci pourrait expliquer cette interaction entre l’évolution de l'IMC et la taille de ménage. Ainsi donc nous recommandons que cet aspect des choses qui n'a pas fait l'objet de notre étude puisse être étudié dans le futur. Le traitement antirétroviral a largement contribué à la réduction de la morbimortalité liée au VHI dans le monde en général et dans les pays à ressources limitées en particulier. Néanmoins, ce traitement reste à vie, et sa prise à vie expose à des complications et contraintes, celles-ci tendent à s'alléger grâce aux progrès de la recherche de traitements plus commodes donnant des résultats satisfaisants et conduisant à la réduction des effets secondaires et des résistances. Notre étude comporte quelques limites, notamment l'absence de marqueurs immunologiques et virologique qui permettraient que des patients avec échec virologique parmi ceux qui présenteraient des marqueurs d'une bonne évolution clinique et immunologique soient identifiables [22]. En dépit du fait que le dosage de la charge virale est devenu beaucoup plus accessible suite à la généralisation du PCR dans les milieux à faibles ressources [22, 23], en RD Congo cette réalité est encore loin d’être à la portée des patients à faible ou même à moyen revenu.

L'absence des marqueurs virologiques et immunologiques crée un frein à l'identification des couples discordants [28, 29] chez qui le suivi devrait être redynamisé. Messou E et al [22], ont observé lors d'une étude sur le succès virologique qu'en cas d'absence de la charge virale, les marqueurs cliniques et immunologiques seuls ne peuvent prédire le succès virologique; bien plus lorsque la charge virale est indétectable au dosage chez les patients avec un taux de CD4 et un IMC bas par rapport aux seuils normaux, à élargir le spectre de recherche de la cause plutôt que de se focaliser à la mauvaise adhérence au traitement comme cause. Nos résultats veulent inciter à tirer plusieurs sonnettes d'alarmes dans le cadre de la lutte contre le sida et de l'amélioration de la condition optimum de traitement ARV afin d'obtenir des résultats satisfaisants à long terme dans le milieu à ressources limitées. Premièrement, ils plaident pour un renforcement de la prévention du sida à large échelle avec insistance sur les groupes cibles notamment la population de moins de 50 ans, les jeunes adolescents et les femmes. Il s'avère aussi opportun d'attirer l'attention des praticiens sur les facteurs ci-haut identifiés qui affaiblissent le maintien des patients dans le circuit des soins, et de développer des stratégies visant à augmenter la rétention des patients déjà mis sous traitement. Après une décennie de passage au traitement ARV à large échelle dans les milieux à ressources limitées, il est important d'investir sur le maintien de respect des conditions de suivi clinique, biologique (immunologique et virologique) et sur l'amélioration des conditions socioéconomiques des patients dans le but de favoriser une bonne observance, une adhérence et limiter la probabilité de survenue des résistances au traitement. Deuxièmement, ces résultats plaident pour un début de traitement ARV à un bon moment, quand le patient est au stade débutant de la maladie en bonne santé pour le maintenir en bon état nutritionnel et avec une vie de bonne qualité. Enfin ces résultats à l'instar d'autres plaident pour des interventions visant à supplémenter nutritionnellement les patients carencés ou ceux issus de niveaux socioéconomiques pauvres dans les pays à ressources limitées notamment en RD Congo.

## Conclusion

Cette étude présente une image épidémiologique, clinique et anthropométrique dans le contexte du traitement ARV à large échelle dans un milieu à ressources limitées en l'occurrence RD Congo. Nous avons observé des facteurs qui affaiblissent le maintien des patients dans les circuits des soins notamment dans le suivi des ARV sur lesquels il faut redynamiser les efforts. Et le taux de rétention des patients sous ARV a été relativement élevé pour ce genre de contexte. Les catégories sociodémographiques, cliniques et anthropométriques où l'on observait les proportions élevées des PPVIH du milieu congolais ont été identifiées. Une évolution faible mais progressive de l’état nutritionnel a été observée, permettant de percevoir l'effet positif de la prise en charge thérapeutique bien qu'il soit encore très difficile de trancher sur le succès virologique et l'adhérence au traitement. Bien que les patients avec IMC bas restent le groupe des patients les plus vulnérables nécessitant une prise en charge thérapeutique rapide et un accompagnement psychosocial et nutritionnel soutenu, nous recommandons pour les recherches futures, des études d'intervention intégrant les aspects ci-dessus et un suivi des marqueurs biologiques qui permettrait d’évaluer aussi bien le succès immunologique, virologique et anthropométrique, qui permettrait aussi de redynamiser la prise en charge thérapeutique dans les milieux à ressources limitées et congolais en particulier.
